# Differential Effects of Lifestyle Indicators on Cognitive Functioning Across Healthy, Dementia, Parkinson’s Disease, and Stroke Groups

**DOI:** 10.3390/jcm15072620

**Published:** 2026-03-30

**Authors:** Adrián García-Mollá, Amparo Oliver, José M. Tomás

**Affiliations:** Department of Methodology of the Behavioral Sciences, University of Valencia, 46010 Valencia, Spain; adrian.garcia-molla@uv.es (A.G.-M.); amparo.oliver@uv.es (A.O.)

**Keywords:** cognitive aging, lifestyle factors, measurement invariance, dementia, Parkinson, stroke

## Abstract

**Background/Objectives**: As life expectancy increases, chronic diseases have become more prevalent, often leading to poorer health in later years. Maintaining cognitive functioning is therefore essential for preserving independence in older adulthood. Within the framework of cognitive enrichment, research highlights the protective role of healthy lifestyles and engagement in social and intellectual activities on cognitive functioning. This study aimed to provide evidence of the moderator effect of diagnosis group (including healthy condition, dementia, Parkinson’s, and stroke) on a predictive model of cognitive function. **Methods**: Data employed in this study came from the 9th wave of the Survey of Health, Ageing and Retirement in Europe (SHARE) project, including 17,105 individuals aged 50 years and older from 27 European countries. Cognitive functioning was assessed through numeracy, temporal orientation, verbal fluency, and memory. Physical inactivity, social participation, intellectual activities, age, gender, and education were included as predictors. A measurement invariance routine across diagnostic groups was tested. **Results**: The model demonstrated excellent fit in the general sample and partial invariance across groups. Physical inactivity was negatively associated with numeracy in all groups, with stronger effects in clinical populations, particularly stroke and dementia. Intellectual activities were positively associated with numeracy across groups, with the largest effects observed in dementia. Temporal orientation, physical inactivity and intellectual activities showed significant associations mainly in clinical groups, whereas age demonstrated a consistent negative effect across all groups. **Conclusions:** Lifestyle factors show differential associations with cognitive domains depending on diagnostic condition. These findings support the heterogeneity of cognitive aging and highlight the importance of tailored, person-centered intervention strategies.

## 1. Introduction

The confluence of declining birth rates and increased life expectancy has led to population aging being positioned as one of the central issues facing health systems currently [1,2]. While birth rates are declining, improvements in healthcare systems and social support, political and economic stability, and the promotion and adoption of healthy lifestyles have led to increased longevity among the global population [3,4]. However, this increase in longevity has also been linked to poorer health during the final years of life, increasing the likelihood of developing chronic diseases such as cardiovascular disease, cancer, or neurodegenerative disorders [5]. Consequently, there is currently a high comorbidity of chronic diseases among older adults, which leads to a decline in quality of life and hinders independent functioning [6,7]. Moreover, the incidence of chronic health conditions constitutes the primary cause of mortality, particularly among the older population [4,5,6,7].

In this sense, aging can be interpreted as a difficult achievement given the prolongation of life expectancy and, at the same time, as a challenge to be overcome by health care and economic systems [4]. For this reason, the most widely accepted definitions of healthy aging focus on preserving the abilities that enable individuals to function independently in their environment and achieve well-being [8]. In particular, maintaining cognitive functioning enables individuals to interact with the environment properly and preserve their quality of life [9,10].

Cognitive changes during old age could represent both a normative aging process and a clinical or pathological performance [11,12]. The first one is characterized by a decline in complex tasks that require attention, visuospatial, speed information processing and some aspects of memory and executive functions [13,14]. On the contrary, cognitive changes in older adulthood may also indicate a pathological trajectory, progressing from Mild Cognitive Impairment (MCI) to dementia. MCI is widely recognized as an intermediate stage preceding dementia, characterized by cognitive symptoms that begin to noticeably interfere with everyday functioning [15,16,17]. Dementia, in turn, involves more pronounced cognitive deterioration, is associated with poorer mental health outcomes, and represents one of the primary causes of disability worldwide [18,19]. According to current literature, both cognitive decline and dementia have been shown to have a substantial impact on an individual’s ability to perform activities of daily living. These activities, which may include instrumental tasks such as using the telephone, preparing meals, or scheduling medical appointments, as well as basic self-care activities like bathing or dressing, are all significantly compromised by these conditions [20,21,22,23].

For its part, other health conditions that could arise suddenly, such as Parkinson’s disease and stroke, may result in the impairment of several or some cognitive domains. Regarding Parkinson’s disease, it constitutes the second most common neurodegenerative disorder, and total cases worldwide are expected to increase from 11.9 million in 2021 to 25.2 (95% CI 21.7–30.1) million in 2050 [24,25]. Although Parkinson’s disease is characterized by impaired motor skills, research indicates that patients with Parkinson’s have significantly poorer cognitive performance than the general population, and that poorer cognitive performance is associated with worse scores on postural instability and gait difficulty [26]. Cognitive impairment in this segment of the population occurs more probably and critically in advanced stages of the disease, in older individuals and appears abruptly [27,28]. Lower performance of tasks involving executive functions, language and memory is usually found among these individuals, along with a subjective feeling of slowed thinking [29,30,31,32].

Regarding stroke, it consists of a cerebrovascular event characterized by the blood flow obstruction to a region of the brain [33]. In this case, prevalence rates range from around 3% among young adults and an estimated 7.7% among individuals older than 65 years old [34]. After a stroke, it is really hard to predict the degree of impairment and the cognitive domains affected, as this will depend on the location of the lesion, the size of the directly affected tissue and the extent of damage beyond the ischemic core. A major part of cognitive symptoms after a stroke event is related to executive function and language-communication abilities, in cases where frontal or temporal areas are affected [35]. However, visuospatial and perceptual-motor functions are involved when the parietal area is affected [36,37]. In addition to cognitive symptoms, mood is also affected by the appearance of anxiety and depressive symptoms after the stroke [38].

The heterogeneity observed across diagnostic conditions reflects the broader reality that cognitive decline is shaped by a complex interplay of biological vulnerability, health status, and lifestyle-related factors. Several risk factors have been associated with worse cognitive trajectories, including environmental exposures [39], cardio-metabolic conditions such as cardiovascular disease, diabetes, and obesity [40], and modifiable behaviors such as physical inactivity or substance misuse [41]. Conversely, engagement in intellectually stimulating and socially meaningful activities has been identified as protective against cognitive deterioration [42,43]. Educational attainment also plays a critical role, contributing to cognitive reserve and mitigating the clinical expression of pathological cognitive decline [44,45,46]. Importantly, these factors do not affect all individuals equally. Different health conditions interact with lifestyle and sociodemographic characteristics to produce distinct patterns of cognitive impairment [47]. For example, vascular risk factors may disproportionately influence executive functioning in stroke survivors, whereas intellectual engagement may exert stronger protective effects in neurodegenerative conditions [48]. To understand these protective or risk effects, it is important to note that, while brain maintenance refers to the preservation of physical integrity, cognitive reserve implies the maintenance of functioning. In this sense, the cognitive enrichment framework provides a mechanistic bridge between lifestyle and these constructs [49].

Considering variability in cognitive trajectories and the distinct patterns associated with different diagnostic groups, standardized treatment approaches may be insufficient. Increasingly, clinical guidelines advocate for person-centered care models that tailor interventions to the individual’s cognitive profile, health status, lifestyle, and personal goals [49]. This type of orientation requires deep knowledge of individual characteristics and the context in which patients are situated [50,51]. As person-centered treatment is defined in the literature, it must not only be based on knowledge of individual characteristics, but a joint decision-making process must also be established among patients, formal caregivers, and their families [52,53]. In this sense, individualized treatment in cases of pathological cognitive decline allows for an improvement in specific skills in order to achieve the most independent daily functioning possible, as stated in previous studies [54]. Likewise, this type of treatment has been shown to reduce symptoms of agitation in people with dementia, which considerably improves their quality of life [55]. In this regard, from the perspective of cognitive enrichment, it is postulated that the presence of individuals in stimulating environments not only correlates with better performance but also facilitates processes related to neural plasticity, such as increased synaptic density [56,57].

In this study, we aimed at examining the potential moderator effects of diagnostic groups on the explanatory effects of lifestyle and sociodemographic indicators on specific cognitive domains. By doing so, this research moves beyond examining main effects to test whether diagnostic status moderates these well-established associations. After establishing the model for the general sample using data from the most recent release of the SHARE project, we will further explore the measurement invariance across normative, dementia, Parkinson and stroke groups. Lifestyle effects are expected to differ between groups due to the higher sensitivity of clinical groups in front of normative conditions. Additionally, the supervening nature of stroke condition leads us to believe that this population may be more predisposed to recovery than degenerative groups, such as those diagnosed with dementia and Parkinson’s disease.

## 2. Materials and Methods

### 2.1. Sample and Procedure

The sample employed in this study comes from the most recent wave (9th) of the Survey of Health, Ageing and Retirement in Europe [58,59]. SHARE is a longitudinal panel study that collects data about physical and mental health, social networks, retirement and income from individuals aged 50 years old or older and their partners, irrespective of their age. Data collection of the 9th wave took place between 2021 and 2022; thus, the present work constitutes a retrospective study.

This study aimed to examine the differential functioning of the diagnostic category on the predictive model estimated in a previous study [60]. Consistently, the inclusion criteria were met if participants were 50 years or older, and they were required to present a diagnosis included in one of the following four categories: Alzheimer’s disease/senility/dementia, Parkinson’s disease, have presented a stroke or were healthy. The final sample for this study consisted of 17,105 individuals representing 27 European countries, 45.9% of them were males and 54.1% were females. The mean age was 66.88 (SD = 9.65), ranging between 50 and 102 years old. The normative group comprised 13,466 individuals, the dementia group comprised 908 individuals, the Parkinson’s group comprised 453 individuals, and the stroke group comprised 2278 individuals.

### 2.2. Instruments

Four cognitive measures were considered: numeracy, temporal orientation, memory and verbal fluency.

Numeracy consisted of a series of five serial subtractions, where individuals were asked to subtract 7 at a time starting from 100. The final score was composed of the accumulation of correct answers, ranging from 0 to 5. This task was corrected so that errors were not cumulative, taking the last number expressed by individuals for the subsequent subtraction. This task involves not only arithmetic processing but also executive-related functions such as working memory and sustained attention. Therefore, performance in this task should be interpreted as reflecting a combination of basic numerical manipulation and executive control processes rather than pure arithmetic ability. It is part of the Mini-Mental State Examination (MMSE) [61].

Temporal orientation was assessed on the basis of four items related to date, month, year and day of week at the moment of the interview. The final score consisted of the accumulation of correct answers, which ranged from 0 to 4. This task was also extracted from MMSE [61].

Memory was assessed employing a 10-word recall test [61]. Participants were asked to recall twice a randomly assigned list of ten words that was read to them. The first recall took place immediately after the list was read (recent recall), and the second recall took place after a delayed time (delayed recall). Two scores were yielded from this task, ranging from 0 to 10, representing the sum of correct answers per set.

Verbal fluency was assessed, requiring the participants to mention the maximum number of animals as possible in one minute, involving the executive function, language and semantic memory [62,63]. The final score represented the number of correctly retrieved animals.

In relation to lifestyle and behavioral indicators, two variables were calculated. These variables were derived from the different kinds of activities that individuals participated in during the previous year. The two variables were participation in social activities and participation in intellectual activities. The first one summarized the scores of volunteerism, attending educational/training courses, joining sport/social/other clubs and taking part in political/community-related organizations, ranging from 0 to 4. And the second one, reading books/magazines/newspapers, doing word or number games and playing cards/chess/similar, ranging from 0 to 3.

Finally, physical inactivity was assessed by asking participants to report the frequency with which they engaged in moderate-intensity activities, such as gardening, car washing, or walking. Responses were collected using a 4-point Likert scale, ranging from 1 (More than once a week) to 4 (‘Hardly ever, or never’). Higher scores on this scale represent greater levels of sedentary behavior. Age, gender (0 = female, 1 = male) and years of education were also included in the model.

### 2.3. Statistical Analysis

First, descriptive statistics of the variables were calculated with SPSS 29 for each of the four diagnosis groups. Then, a predictive model of cognitive functioning was established following the results of previous studies employing data from the last wave of the SHARE project [58,59]. On this occasion, the model was established to include age, gender, years of education, physical inactivity, participation in social activities, and participation in intellectual activities as antecedents for cognitive function. However, chronic diseases were excluded because they were employed to establish diagnosis groups. Regarding cognitive functioning, it was measured by temporal orientation, numeracy, verbal fluency and memory, which was established as a latent variable that summarized scores of recent and delayed recalls. Therefore, the estimated model was a Multiple Indicators, Multiple Causes model (MIMIC). The election of the MIMIC model over other statistical techniques is determined by the nature of the variables/indicators and the aims of the manuscript. Firstly, the variables that integrate the model are observed indicators that cannot be modeled with latent variables (age, gender, years of education, physical inactivity, social activities, intellectual activities, numeracy, temporal orientation and verbal fluency). The only exception is memory, with two indicators (recent and delayed records) that can be modeled as a latent variable. Secondly, the model is a purely predictive model of several predictors on the cognitive measures. For the estimation, Mplus 8.11 [64] was employed. Given the nonnormality of the data, the estimator was Maximum Likelihood Robust (MLR) [65,66]. Missing data were dealt with Full Information Maximum Likelihood (FIML) [67]. Several fit indexes were employed to assess the fit of the proposed model on the general sample and to compare the nested models throughout the invariance routine: the chi-square statistic (χ^2^), the Comparative Fit Index (CFI), the Standardized Root Mean Square Residual (SRMR), and the Root Mean Squared Error of Approximation (RMSEA). Adequate model fit was established with CFI values of 0.90 or higher and SRMR and RMSEA values below 0.08 [68].

Once the model for the general sample was estimated, we followed a conventional measurement invariance procedure [69]. First, an unconstrained configural model was estimated. Next, metric invariance was examined by imposing equality constraints on factor loadings across diagnosis groups. Subsequently, scalar invariance was tested by constraining both factor loadings and item intercepts to be equal across diagnosis groups. Then, in order to study the moderator effect of diagnosis group on variable relationships, modification indexes were taken into account to remove parameter restrictions one by one until an optimal adjustment level is achieved. For the traditional invariance testing sequence, we considered changes in the Comparative Fit Index (ΔCFI) smaller than 0.01 between nested models as evidence of no meaningful decline in model fit, supporting the more constrained and parsimonious specification [70].

## 3. Results

### 3.1. Descriptive Statistics

The data showed distinct profiles across the four study groups regarding demographics and lifestyle habits. The normative group was the youngest on average (M = 64.83, SD = 8.62) and had the highest educational attainment (M = 12.18 years, SD = 3.96). Women represented 55.1% of this group. This group also showed the most active lifestyle profile: nearly three-quarters engaged in physical activity more than once a week (72.8%), and they reported the highest participation in social and intellectual activities. Although 61.2% reported no social activities, this percentage was markedly lower than in the clinical groups, and they displayed the highest proportions of engagement in multiple intellectual activities, particularly at the level of three activities (17.1%).

The dementia group was the oldest (M = 77.85, SD = 9.16) and had the lowest average years of education (M = 10.3, SD = 4.28). Women comprised 57.7% of this group. This group showed the lowest levels of engagement across all activity domains. Only 41.7% reported engaging in physical activity more than once a week, while 37.4% reported hardly ever or never being physically active. Social inactivity was especially pronounced, with 84.8% reporting no social activities. Intellectual engagement was also reduced, with the highest proportion reporting no intellectual activities (39%) and the lowest percentage participating in three activities (8.9%).

The Parkinson group had a mean age of 75.36 years (SD = 8.50) and an average of 10.72 years of education (SD = 4.75). Women accounted for 49.4% of the group. Physical activity levels were intermediate between the normative and dementia groups, with 51.4% engaging more than once a week and 30.5% reporting little or no activity. Social participation was limited, with 77.3% reporting no social activities. Intellectual engagement appeared somewhat better preserved compared to the dementia group, with relatively balanced participation across one and two activities (27.4% and 29.6%, respectively), although participation in three activities remained modest (12.6%).

Finally, the stroke group had a mean age of 72.95 years (SD = 9.38) and an average of 10.99 years of education (SD = 3.99). Women represented 47.9% of the group, the lowest proportion among the four groups. Physical activity was reduced compared to the normative group, with 51.1% engaging more than once a week and 28.2% reporting hardly ever or never being active. Social inactivity was high (76.3%), though slightly lower than in the dementia and Parkinson groups. Intellectual activity levels were relatively similar to the Parkinson group, with a moderate distribution across one (28.7%) and two activities (26.2%), but lower engagement in three activities (13.1%) compared to the normative group. Descriptive statistics for the preceding indicators and the cognitive scores of each group are presented in Table 1 and Table 2, respectively.

### 3.2. Structural Model and Measurement Invariance

**Figure 1:**
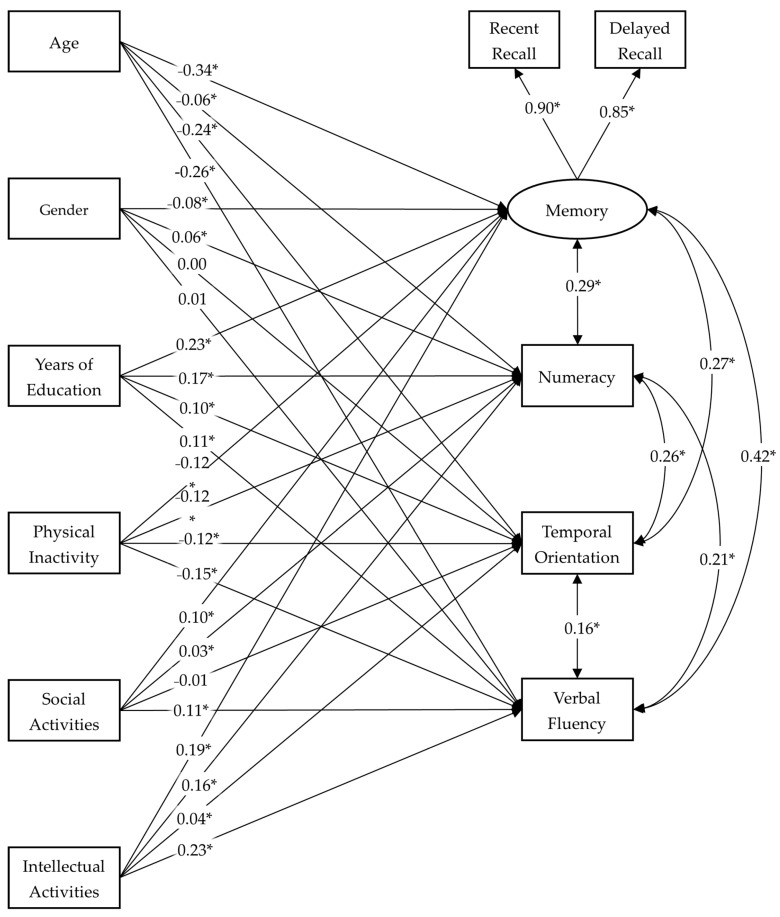
Standardized parameters for the model fitted to the general sample. Note: * = *p* < 0.01. Gender coded 0 = female, 1 = male.

First, the MIMIC model was estimated on the general sample. Fit indexes for the model showed excellent fit to the data: χ^2^ (8) = 93.941, *p* < 0.001, CFI = 0.997, RMSEA = 0.025, [90% CI 0.021–0.030] and SRMR = 0.004. Standardized parameters of the general sample model are shown in Figure 1, and correlation coefficients among exogenous variables are displayed in Table 3.

After the model was established and estimated on the general sample, the results of estimating configural, metric and scalar invariance indicate partial measurement invariance across diagnosis groups. Nevertheless, the estimated models demonstrated an adequate fit to the data. Fit indexes for the conventional invariance routine are displayed in Table 4.

The moderating effect of the group on the relationships between the variables in the model was studied by sequentially removing the parameter restrictions indicated in the modification indices. Therefore, the first effect that was left free to estimate was the numeracy coefficient on intellectual activities. Subsequently, the coefficient of temporal orientation on physical inactivity, the coefficient of temporal orientation on intellectual activities, the coefficient of numeracy on physical inactivity, and, finally, the coefficient of temporal orientation on age. This last model was retained for subsequent analysis of the differentiation of effects across diagnostic groups, given that the fit indices show a good approximation to the fit of the metric model of the invariance routine. The fit indices for the sequential removal of parameter restrictions from the scalar model are presented in Table 5.

Regarding the free estimated coefficients, physical inactivity was negatively associated with arithmetic performance in all groups, although the magnitude of the association varied considerably. The effect was smaller in the normative group, while stronger negative associations were observed in the clinical groups, especially among people with dementia and stroke, followed by people with Parkinson’s disease.

In contrast, intellectual activities showed a positive association with arithmetic in all groups, with notably greater effects in clinical populations. The strongest association was observed in the dementia group, while the Parkinson’s and stroke groups also showed stronger effects than the normative group, suggesting that participation in intellectually stimulating activities may be particularly relevant for maintaining calculation ability in people with neurological conditions.

For temporal orientation, the pattern was different. Physical inactivity was not significantly related to orientation in the normative group but showed significant negative associations in all clinical groups, again with the strongest effect in the dementia group.

The association between intellectual activities and temporal orientation was comparatively lower and more diagnosis-specific. In the normative and Parkinson groups, the relationship was not statistically significant. By contrast, it was significant in the dementia and stroke groups, although the magnitude remained modest. This pattern suggests that intellectual activities are more strongly associated with temporal orientation in conditions characterized by greater cognitive impairment.

Finally, age was negatively associated with temporal orientation across all groups, with highly significant relations (all *p* < 0.001). The magnitude of the association was relatively similar in the normative, dementia, and Parkinson groups, indicating a consistent age-related decline in orientation abilities regardless of diagnosis. The association was somewhat weaker in the stroke group. Overall, age appears to exert a robust and broadly comparable influence on temporal orientation across diagnostic categories, whereas lifestyle-related factors show more pronounced variability in their impact. The freely standardized coefficients in each group are shown in Table 6.

## 4. Discussion

In accordance with the theoretical framework of cognitive enrichment, several researchers have proposed the hypothesis that engagement in social and intellectual activities fosters the preservation of cognitive functioning during adulthood and in late life [70,71,72,73].

This study aimed to examine the associations of lifestyle, operationalized as physical inactivity and participation in social and intellectual activities, on cognitive function in the most recent data from older adults in the SHARE project, specifying the domains of memory, arithmetic, temporal orientation, and verbal fluency. The specification of this model comes from previous research. The main objective of this study was to test the moderating effects of the diagnostic group on the effects of background factors within this predictive model of cognitive functioning, differentiating between patients without a diagnosis, patients with dementia, patients with Parkinson’s disease, and patients who had suffered a stroke.

Results from this study show that the predictive model of cognitive functioning had an excellent fit in the general sample and achieved partial measurement invariance across diagnostic groups [69], indicating that while the overall cognitive structure was comparable, several structural relationships differed meaningfully between groups. Descriptive results revealed clear demographic and lifestyle differences: the normative group was younger, more educated, and more actively engaged in physical, social, and intellectual activities, whereas the dementia group was the oldest and least active across domains. Parkinson’s and stroke groups showed intermediate profiles, though both presented reduced activity engagement compared to healthy individuals.

Regarding the associations of predictors on cognitive measures, lifestyle factors exhibited differential effects across diagnoses. Physical inactivity was negatively associated with numeracy in all groups, but its impact was substantially stronger in the clinical group, particularly in stroke and dementia, compared to the normative group. In this line, the previous evidence indicates the effectiveness of physical-activity interventions on stroke, Parkinson’s, and dementia [74,75,76]. Similarly, intellectual activities were positively associated with numeracy across all groups, yet the magnitude of this association was markedly larger among individuals with dementia, followed by Parkinson’s and stroke patients. This suggests that intellectual engagement may play a more pronounced compensatory or protective role in clinical populations. In this line, cognitive training interventions also showed evidence in maintaining cognitive performance in individuals with dementia, stroke, and Parkinson’s disease [77,78,79].

For temporal orientation, the pattern was more diagnosis-specific. Physical inactivity showed no significant association in the normative group but demonstrated significant negative relations in all clinical groups, especially among individuals with dementia. Intellectual activities were unrelated to temporal orientation in healthy and Parkinson groups, yet showed modest but significant positive associations in dementia and stroke groups. In contrast, age exerted a robust and consistent negative association with temporal orientation across all groups, with only slightly weaker effects in the stroke group.

Overall, the results indicate that although age-related cognitive decline follows a relatively stable pattern across diagnostic categories, the influence of modifiable lifestyle factors varies substantially depending on the underlying health condition. Clinical groups appear more sensitive to both the negative association of physical inactivity and the positive association of intellectual engagement, supporting the notion that cognitive aging is not homogeneous but condition-specific. However, this study also presented certain limitations. First, the use of cross-sectional data does not really allow for the study of causal relationships, since the principle of precedence is not fulfilled. It limited the study to draw exploratory conclusions about the relationships between the variables, and it does not guarantee the existence of a possible reverse effect or bidirectional relationship. Future research lines should be focused on the study of the directionality of effects by using longitudinal data. Additionally, the predictor measures had some missing data given the length of the interview and the large number of variables to be evaluated, which is common in this type of data. On the other hand, cognitive functioning measures could present ceiling effects on the healthy group as they are derived from the MMSE, which has been demonstrated to discriminate better on clinical populations [80]. Lifestyle indicators are based solely on a count of activities participated in during the past year, without distinguishing between subjects based on frequency of participation. Lastly, the categorical variable employed to create diagnosis clusters could be more specific to the response to dementia, as it includes every kind of dementia diagnosis or senile condition in the same group. Similarly, the group diagnosed with stroke does not take into account the variability that could exist depending on the severity of the case due to response time, extent, and affected areas. This could lead to differences in the impact on specific domains [57]. Additionally, a limitation of the structural model (and by extension of most predictive models) is the analytical difficulties to handle a large number of potentially confounding variables, such as disease severity and duration; medication effects; socioeconomic status; depression; cardiovascular risk; functional status; and so on and so forth. This must be acknowledged as a limitation of the current study.

## 5. Conclusions

The present study underscores the heterogeneous nature of cognitive aging by demonstrating that the associations between lifestyle factors and cognitive performance vary meaningfully across diagnostic groups. While age showed a consistent negative relationship with temporal orientation across all participants, the effects of physical inactivity and intellectual engagement were substantially stronger in clinical populations, particularly among individuals with dementia and stroke. These findings suggest that modifiable lifestyle factors may play a more pronounced role in shaping cognitive outcomes when underlying neurological conditions are present. Overall, the results reinforce the need to move beyond a uniform understanding of cognitive decline and instead adopt differentiated, person-centered approaches that account for diagnostic status, health profiles, and individual engagement patterns when designing prevention and intervention strategies.

## Figures and Tables

**Table 1:** Descriptive statistics of the indicators under study.

Indicators	Mean ± SD or n (%)			
	Normative	Dementia	Parkinson	Stroke
Age	64.83 ± 8.62	77.85 ± 9.16	75.36 ± 8.50	72.95 ± 9.38
Gender (women)	7421 (55.1%)	524 (57.7%)	224 (49.4%)	1092 (47.9%)
Years of education	12.18 ± 3.96	10.3 ± 4.28	10.72 ± 4.75	10.99 ± 3.99
Physical inactivity				
More than once a week	9800 (72.8%)	379 (41.7%)	233 (51.4%)	1164 (51.1%)
Once a week	1876 (13.9%)	118 (13.0%)	48 (10.6%)	308 (13.5%)
One to three times a week	874 (6.5%)	71 (7.8%)	34 (7.5%)	164 (7.2%)
Hardly ever. or never	914 (6.8%)	340 (37.4%)	138 (30.5%)	642 (28.2%)
Missing	2 (0.0 14%)	-	-	-
Social Activities				
No activity	8241 (61.2%)	770 (84.8%)	350 (77.3%)	1738 (76.3%)
One activity	3239 (24.1%)	94 (10.4%)	72 (15.9%)	394 (17.3%)
Two activities	1447 (10.7%)	31 (3.4%)	25 (5.5%)	111 (4.9%)
Three activities	450 (3.3%)	9 (1%)	5 (1.1%)	27 (1.2%)
Four activities	80 (0.6%)	2 (0.2%)	1 (0.2%)	5 (0.2%)
Missing	9 (0.1%)	2 (0.2%)	-	3 (0.1%)
Intellectual activities				
No activity	3657 (27.2%)	354 (39%)	138 (30.5%)	725 (31.8%)
One activity	3816 (28.3%)	263 (29%)	124 (27.4%)	654 (28.7%)
Two activities	3681 (27.3%)	208 (22.9%)	134 (29.6%)	597 (26.2%)
Three activities	2303 (17.1%)	81 (8.9%)	57 (12.6%)	299 (13.1%)
Missing	9 (0.1%)	2 (0.2%)	-	3 (0.1%)

**Table 2:** Descriptive statistics of the cognitive scores per group.

Indicators	Mean ± SD or n (%)			
	Normative	Dementia	Parkinson	Stroke
Recent Recall	5.7 ± 1.71	3.23 ± 1.98	4.31 ±1.73	4.53 ± 1.82
Delayed Recall	4.33 ± 2.13	1.58 ± 1.9	2.64 ± 1.97	2.98 ± 2.1
Verbal Fluency	22.48 ± 8.37	13.10 ± 7.23	16.93 ±7.53	17.95 ± 7.9
Numeracy				
No correct answers	91 (0.7%)	60 (6.6%)	10 (2.2%)	51 (2.2%)
One correct answer	450 (3.3%)	129 (14.2%)	44 (9.7%)	215 (9.4%)
Two correct answers	414 (3.1%)	77 (14.2%)	31 (6.8%)	146 (6.4%)
Three correct answers	1032 (7.7%)	91 (10%)	43 (9.5%)	279 (12.2%)
Four correct answers	1733 (12.9%)	115 (12.7%)	58 (12.8%)	335 (14.7%)
Five correct answers	9543 (70.9%)	296 (32.6%)	232 (51.2%)	1131 (49.6%)
Missing	203 (1.5%)	140 (15.4%)	35 (7.7%)	121 (5.3%)
Temporal Orientation				
No correct answers	27 (0.2%)	90 (9.9%)	-	25 (1.1%)
One correct answer	44 (0.3%)	70 (7.7%)	10 (2.2%)	35 (1.5%)
Two correct answers	96 (0.7%)	115 (19.3%)	18 (4%)	71 (3.1%)
Three correct answers	739 (5.5%)	175 (19.3%)	69 (15.2%)	309 (13.6%)
Four correct answers	12,560 (93%)	453 (49.9%)	356 (78.6%)	1838 (80.7%)
Missing	-	5 (0.6%)	-	-

**Table 3:** Correlation coefficients among exogenous variables. All correlations are statistically significant (*p* < 0.05).

	(1)	(2)	(3)	(4)	(5)
(1) Age	1				
(2) Gender	0.052	1			
(3) Years of education	−0.172	−0.020	1		
(4) Physical inactivity	0.232	−0.035	−0.158	1	
(5) Social activities	−0.131	0.006	0.255	−0.231	1
(6) Intellectual activities	−0.019	−0.080	0.217	−0.268	0.342

**Table 4:** Model fit results for the conventional invariance routine.

Model	χ^2^	df	*p*	RMSEA	90% CI	SRMR	CFI	∆CFI
Configural	213.911	35	<0.01	0.035	0.030–0.039	0.009	0.993	
Metric	232.380	38	<0.01	0.035	0.030–0.039	0.011	0.993	0.00
Scalar	654.081	110	<0.01	0.034	0.032–0.037	0.031	0.980	0.013

**Table 5:** Model fit results for the sequential parameter restrictions removal.

Model	χ^2^	df	*p*	RMSEA	90% CI	SRMR	CFI	∆CFI
Scalar	654.081	110	<0.01	0.034	0.032–0.037	0.031	0.980	
Model 1	531.897	107	<0.01	0.030	0.028–0.033	0.026	0.984	−0.004
Model 2	426.917	104	<0.01	0.027	0.024–0.030	0.020	0.988	−0.004
Model 3	388.663	101	<0.01	0.026	0.023–0.029	0.018	0.989	−0.001
Model 4	368.129	98	<0.01	0.025	0.023–0.028	0.017	0.990	−0.001
Model 5	347.437	95	<0.01	0.025	0.022–0.028	0.017	0.991	−0.001

**Table 6:** Free estimated standardized coefficients for each diagnosis group.

Indicators	Standardized Coefficients (*p*-Value)			
	Normative	Dementia	Parkinson	Stroke
Numeracy				
Physical inactivity	−0.049 (<0.001)	−0.132 (<0.001)	−0.118 (0.007)	−0.135 (<0.001)
Intellectual activities	0.139 (<0.001)	0.327 (<0.001)	0.243 (<0.001)	0.245 (<0.001)
Temporal orientation				
Physical inactivity	−0.017 (0.138)	−0.182 (<0.001)	−0.140 (0.002)	−0.137 (<0.001)
Intellectual activities	0.009 (0.354)	0.122 (<0.001)	0.057 (0.175)	0.091 (<0.001)
Age	−0.188 (<0.001)	−0.179 (<0.001)	−0.169 (<0.001)	−0.108 (<0.001)

## Data Availability

The data that support the findings of this study are available from the SHARE project, but restrictions apply to the availability of these data, which were used under license for the current study, and so are not publicly available. Data are, however, available from the authors upon reasonable request and with permission of the SHARE Project.
